# Dyadic care to improve postnatal outcomes of birthing people and their infants: A scoping review protocol

**DOI:** 10.1371/journal.pone.0298927

**Published:** 2024-04-16

**Authors:** Courtney C. Choy, Molly E. McAdow, Julia Rosenberg, Alyssa A. Grimshaw, Josefa L. Martinez-Brockman

**Affiliations:** 1 Department of Chronic Disease Epidemiology, Yale School of Public Health, New Haven, Connecticut, United States of America; 2 Department of Obstetrics, Gynecology, and Reproductive Sciences, Yale School of Medicine, New Haven, Connecticut, United States of America; 3 Department of Pediatrics, Yale University School of Medicine, New Haven, Connecticut, United States of America; 4 Harvey Cushing/John Hay Whitney Medical Library, Yale University, New Haven, Connecticut, United States of America; 5 Department of General Internal Medicine, Yale School of Medicine, New Haven, Connecticut, United States of America; Menzies School of Health Research, AUSTRALIA

## Abstract

**Introduction:**

Dyadic care, which is the concurrent provision of care for a birthing person and their infant, is an approach that may improve disparities in postnatal health outcomes, but no synthesis of existing dyadic care studies has been conducted. This scoping review seeks to identify and summarize: 1) dyadic care studies globally, in which the birthing person-infant dyad are cared for together, 2) postnatal health outcomes that have been evaluated following dyadic care interventions, and 3) research and practice gaps in the implementation, dissemination, and effectiveness of dyadic care to reduce healthcare disparities.

**Materials and methods:**

Eligible studies will (1) include dyadic care instances for the birthing person and infant, and 2) report clinical outcomes for at least one member of the dyad or intervention outcomes. Studies will be excluded if they pertain to routine obstetric care, do not present original data, and/or are not available in English or Spanish. We will search CINAHL, Ovid (both Embase and Medline), Scopus, Cochrane Library, PubMed, Google Scholar, Global Health, Web of Science Core Collection, gray literature, and WHO regional databases. Screening will be conducted via Covidence and data will be extracted to capture the study design, dyad characteristics, clinical outcomes, and implementation outcomes. The risk of bias will be assessed using the Joanna Briggs Institute Critical Appraisal Tool. A narrative synthesis of the study findings will be presented.

**Discussion:**

This scoping review will summarize birthing person-infant dyadic care interventions that have been studied and the evidence for their effectiveness. This aggregation of existing data can be used by healthcare systems working to improve healthcare delivery to their patients with the aim of reducing postnatal morbidity and mortality. Areas for future research will also be highlighted.

**Trail registration:**

This review has been registered at Open Science Framework (OSF, https://osf.io/5fs6e/).

## Introduction

The postnatal period, often referred to as the “fourth trimester,” is a critical time to support and improve the health outcomes of birthing people and their infants [1, 2]. Pregnancy-related morbidity and mortality often occurs in the 6-week postpartum period, and adverse events during this period are frequently secondary to preventable causes, including depression and suicide, hypertensive disorders, and thromboembolic events [3–5]. Despite these risks, up to half of birthing people in the United States do not attend their visit for postnatal care with a healthcare professional [6]. Health care disparities play a significant role in pregnancy-related morbidity and mortality [7, 8]. In the United States, Black birthing people are three times more likely than White birthing people to die from pregnancy-related complications, and this is partly attributed to difficulties accessing high-quality care [9]. Glazer and colleagues have highlighted that healthcare disparities between the pregnant and postnatal individual and their newborn are interrelated, and the provision of dyadic care may reduce morbidity and mortality risk [10].

While the concurrent provision of care for a birthing person and infant may improve health outcomes, no standard exists for dyadic care either in the United States or globally [11]. A recent (2023) World Health Organization (WHO) postnatal care model places the birthing person-infant dyad at the center of care, with continuous care in the first 24 hours after birth and a minimum of three additional postnatal care contacts [12]. The American Academy of Pediatrics recommends prenatal visits to establish pediatric medical homes and postnatal depression screening for the birthing person during pediatric visits [13]. The American College of Obstetricians and Gynecologists endorses earlier and more expansive postnatal care including assessment of infant care and feeding [14]. Continued postnatal support from family and health services is critical to ensure positive outcomes and requires a combination of supplies, infrastructure, effective referral systems, and respectful, individualized, person-centered care. A key endpoint of the dyadic care model is a ‘positive postnatal experience’ for all birthing people, newborns, parents, caregivers, and families after birth, which will in turn, provide a stronger foundation for improved health outcomes across the life course [12].

The scope and variety of birthing person-infant dyadic care models are not described in societal guidelines. Based on a preliminary search in Embase, no reviews of dyadic care for birthing people and infants were published nor reported as ongoing. Additional searches in PROSPERO and Open Science Framework resulted in no relevant dyadic care model protocols. A synthesis of previously studied models could inform healthcare systems on interventions to reduce pregnancy-related morbidity and mortality.

Here, we describe a scoping review protocol to synthesize original studies and reports that discuss dyadic care and address postnatal health outcomes globally. We will apply the framework described by Glazer and colleagues to define perinatal dyadic care as medical encounters where both the birthing person and the infant receive care at the same time, making it easier for the dyad to receive essential postnatal care [10].

1. We aim to identify dyadic care models that may be replicated and/or adapted for use through the following questions: What models of birthing person-infant dyadic care have been described in published original studies and reports?
2. How were the birthing person and infant dyad characteristics and outcomes measured and defined?
3. What evidence supports the clinical efficacy of dyadic approaches to care?
4. What research knowledge and practice gaps remain in the existing literature for future investigations?

This scoping review will highlight promising practices and future areas for research regarding dyadic postnatal care, which has the potential to reduce healthcare disparities and improve health outcomes.

## Materials and methods

This protocol for the scoping review is shared on the Open Science Framework (https://osf.io/5fs6e/) and aligns with guidelines provided by the Preferred Reporting Items for Systematic Review and Meta-Analysis Protocols (PRISMA-P) Statement (S1 Checklist). When completed, the scoping review will be reported according to guidance from the Preferred Reporting Items for Scoping Review (PRISMA ScR) and Joanna Briggs Institute Manual for Evidence Synthesis [15–19].This scoping review protocol was written in accordance with the Preferred Reporting Items for Systematic Review and Meta-Analysis Protocols (S1 Checklist PRIMSA-P). We will provide details of any amendment made after publication, including, but not limited to, date and description of the change(s) with a rationale in the future.

### Eligibility criteria

Studies will be included if they fulfill the following criteria:

#### Study inclusion and exclusion criteria

Studies will include models of medical care in which both the birthing person (including pregnant and up to 1-year postpartum) and the neonate or infant aged 0 up to 1 year-old receive medical care in the same instance. The instances of dyadic care may include, but are not limited to, pregnancy, postpartum, lactation, and infant care settings. Dyadic interventions during pregnancy will be included if they represent novel approaches to improve neonatal outcomes beyond standard obstetric care. Studies will be excluded if they pertain to routine obstetric management of labor and delivery, quality metrics related to routine obstetric management of labor and delivery, and/or parenting practices without explicit care of the birthing person.

We will use the following screening questions to evaluate inclusion and exclusion criteria:

Include if yes to all:

1. Were the birthing person (including pregnant and up to 1-year postpartum) and neonate/infant (aged 0 to 1 year old) cared for or evaluated in the same encounter?
2. Was an outcome of either the birthing person or the neonate/infant measured or reported (e.g., clinical health, intervention, and/or implementation outcome)?
3. Does the article include original scientific data (e.g., not a review or meta-analysis)?

Exclude if yes:

4. Does the study pertain to obstetric management of labor and delivery and/or quality metrics related to obstetric management of labor and delivery?

#### Publication date

We will include all relevant studies published with no year limitations.

#### Publication types

This scoping review will include both peer-reviewed articles and gray literature to represent the work in the field of dyadic care globally. Conference abstracts, systematic reviews, and meta-analyses will not be included. Conference abstracts will be excluded because studies have found preliminary data often differs from published manuscript data [20, 21].

#### Study setting/context

Studies from all geographic locations will be included. All locations of care delivery will be considered including outpatient visits, inpatient admissions, and home-based care.

#### Language and terminology

Studies published in English and Spanish will be included because one member of the study team (JMB) is fluent in both languages. If the title and abstract are in English or Spanish and it passes title/abstract screening, but the full text is written in a different language, we will search for an English translation. If a translated version cannot be obtained, we will exclude the study in the full-text screen.

#### Terminology considerations

Throughout literature, female-gendered parental terminology (e.g., mother, maternal, breastfeeding) is often used to describe birthing people [22–24]. While our search strategy will include gendered and gender-inclusive terminology, we will use gender-neutral terminology (e.g., birthing person, breast/chestfeeding) in this protocol and future publications.

### Search strategy

We developed a search strategy using controlled vocabulary and keywords that envisioned three potential settings/models where dyadic care may occur: 1) Dyadic models of care (including shared care and appointments); 2) Postpartum and postnatal care encounters (including birthing person/mother, infant/child, postpartum, and postnatal terms); and 3) Birthing person and infant/child services. Our search strategy for Embase is shown (Table 1). Following the Peer Review of Electronic Search Strategies (PRESS) procedure [25], an independent health sciences librarian reviewed our strategy.

**Table 1:** Search strategy in Embase.

Search Results from 12/19/2022
**Dyadic Models of Care** 1 shared medical appointment/ 2 (dyad* adj3 (intervention* or therap* or treatment* or care or healthcare or appointment* or strag* or model* or follow-up* or appointment* or consultation* or visit*)).tw,kf. 3 (dual adj3 (appointment* or care* or healthcare* or consultation* or follow-up*)).tw,kf. 4 ((family or shared) adj3 (appointment* or consultation* or visit* or follow-up*)).tw,kf. 5 or/1-4
**Postpartum and Postnatal Care** 6 exp postnatal care/ 7 exp perinatal care/ 8 exp prenatal care/ 9 kangaroo care/ 10 exp breast feeding/ 11 (after-birth* or afterbirth* or antenatal* or ante-natal or antepartum* or ante-partum* or birth* or breastfeed* or breast-feed* or childbirth* or intranatal* or intra-natal* or intrapartum* or intra-partum* or kangaroo* or lactat* or maternal or maternity or natal* or neonatal* or parenting or parturient* or parturition or perinatal* or post-labor* or post-labour* or preconcept* or pregnan* or post-partum or postpartum or postnatal* or post-natal* or prenatal or pre-natal or puerperal* or puerperant* or puerperium* or trimester*).tw,kf. 12 ((mother* or parent* or father* or caregiver* or maternal or paternal) adj3 (infant* or child* or neonat* or fetus* or fetal* or baby or babies or newborn*)).tw,kf. 13 or/6-12
**Birthing Person & Infant/Child Services** 14 ((care or healthcare or service*) adj3 (mother* or parent* or father* or caregiver* or birthing person* or maternal or paternal or guardian*) adj1 (infant* or child* or neonat* or baby or babies or fetus* or fetal* or newborn*)).ti.
**Combining Concepts** 15 5 and 13 16 14 or 15

#### Information sources

The following nine databases will be searched: CINAHL, Ovid (both Embase and Medline), Scopus, Cochrane Library, PubMed, Google Scholar, Global Health, Web of Science Core Collection to find relevant articles from the inception of the database to the final search date [26]. CitationChaser will be used to find any additional relevant articles not retrieved by database searches that were cited by included studies [27]. The initial search was conducted on December 19, 2022 and according to best practices will be updated within six months of publication and as necessary [19].

In addition to the databases searches, we will manually search Google using the key concepts described above. A study investigator will review the first 10 pages of results retrieved within Google. These searches will be used to retrieve gray literature reports of dyadic care models. Additional site-specific Google searches will be performed for international agencies, such as the World Health Organization.

### Data management

The search results will be preserved in an Endnote 20 (Clarivate Analytics LLC, Philadelphia, PA) file. Screening data will be preserved in Covidence (Covidence, Melbourne VIC Australia)to deduplicate and screen articles. The identification and selection process of articles will be illustrated with a PRISMA flowchart.

The criteria for the exclusion and inclusion of articles were piloted prior to the screening of the title-abstracts. To pilot the criteria, four authors reviewed 50 records and made decisions (either yes or no) to include the title-abstract for full-text screening. If there were disagreements among the authors, their interpretation of the criteria was discussed to achieve a consensus. Inclusion and exclusion criteria were further refined and finalized after discussions with the full authorship team.

The screening process will involve two phases using Covidence. Titles and abstracts for each article will be screened by two independent co-investigators to determine articles for inclusion, for exclusion, or for which the full text is required to determine eligibility. Subsequently, full text screening will be conducted by two independent co-investigators. The investigation team will discuss any disagreements to reach consensus on whether to include or exclude the article. Any reason for exclusion of an article will be documented at full-text screening phase.

#### Data extraction process

We will extract data from all eligible articles. Data extraction and quality assessment results will be collected through Qualtrics and preserved in Excel. Data extraction for each article will be performed by two independent co-investigators with a third independent co-investigator resolving conflicts. Data extraction categories are listed in Table 2. We will use a customized sheet to collect all the relevant information to the aim of this scoping review. This data extraction sheet will be piloted with several articles and modified as necessary to include all the relevant information of the included articles. All study data will be preserved for three years after publication or longer if deemed appropriate by the publishing journal’s data management policy.

**Table 2:** Data extraction categories.

Publication information • Citation year and first author • Type (e.g., original research article, report) •Funding source Study setting •Country location •Data source Study characteristics •Objective(s) •Study design (e.g., observational, randomized control trial, cross-sectional, etc) •Timing (e.g., prenatal, delivery admission, postpartum) •Duration of intervention •Duration of follow-up •Care delivery type (e.g., home visits, outpatient visit) •Implementers of study (e.g., study staff, stakeholders, community health workers, lactation consultants) •Dyadic care model (e.g., shared medical visit, social intervention) •Theoretical model of behavior change, if any •Implementation strategy •Evaluation strategy Dyad characteristics •Sample size (e.g., intervention vs. control groups) •Gestational age of delivered infant •Trimester of pregnancy and/or duration postpartum •Age(e.g., fetus/infant: <0 months, 0–1 month, 1–6 months, >6 months; caregiver age ranges <18, 18–34, ≥35) •Sex assigned at birth •Race and ethnicity •Health status Outcomes •Clinical outcomes (e.g., maternal readmission, hypertensive urgency/emergency, wound complication, thromboembolic disease; pediatric outcomes–growth, development, breastfeeding, safe sleep) •Implementation outcomes (e.g., feasibility, acceptability, cost)

#### Patient and public involvement

To draft and administer this scoping review protocol, patients and the public are not involved. This scoping review will summarize original scientific articles and other article types (e.g., brief research reports) and this may include information about patients and the public.

### Assessment of risk of bias

The Joanna Briggs Institute critical appraisal tools will be used to assess the risk of bias in each included study [28–30]. Studies will be rated by at least two independent reviewers, ratings will be discussed, and discrepancies in ratings will be resolved. Risk of bias ratings will be displayed in the results table. The critical appraisal of the included studies will be used to summarize our confidence in the cumulative evidence.

### Study status and timeline

The gray literature search and screening process are in progress. The search will be re-run at the conclusion of the data extraction and at least six months prior to manuscript submission. We anticipate that this scoping review will be completed by March 2024.

### Presentation of findings

Dyadic care models for birthing people and their infants will be summarized by their characteristics and reported outcomes. Advances in clinical care, as well as gaps in research and clinical practice will be highlighted. Extracted data will be described by country location, study characteristics (e.g., dyadic care model), and quantitative and/or qualitative outcomes. We will develop tables and figures to map and compare the evidence for future research and dyadic care practice and research.

#### Ethics and dissemination

Formal ethical approval is not required for this scoping review that will synthesize existing dyadic care models. For dissemination, we plan to develop a manuscript that includes a comparison and discussion of the identified dyadic care model studies. The findings will be shared through presentations in relevant meetings including those with community groups and peer-reviewed publications.

## Discussion

While standard obstetric and postnatal care have dramatically improved pregnancy outcomes, there remains significant maternal morbidity and mortality, often from preventable causes that disproportionately affect Black birthing people in the United States. Because risk factors for adverse outcomes are shared between the birthing person and infant, dyadic care in which both are cared for together has been proposed as an opportunity to reduce disparities. Existing dyadic care models for birthing people-infant dyads will be identified and summarized in this scoping review. The findings will help to guide and inform healthcare practices and future research to improve the development, implementation, and evaluation of dyadic care models, which are promising opportunities to reduce morbidity and mortality and promote health across the lifespan for both members of the birthing person-infant dyad.

### Strengths and limitations of study design

This scoping review is timely and aligns with WHO’s vision for care across the pregnancy, childbirth, and postnatal continuum [12]. The search strategy was planned to capture a diverse range of dyadic care models in different populations in the U.S. and globally. Considering that the terminology for dyadic care is emerging and inconsistently used in the literature to capture this concept, the search will be broad to find all potentially relevant papers. Our methodology includes efforts to ensure relevant papers not identified by the original search are subsequently identified by searching for grey literature with Google and performing citation chasing; however, it is possible that papers may be missed due to the inconsistency of language used. Our study excludes conference abstracts because they have been found to be unreliable and fail to include adequate information [31–33]. We also chose to focus on birthing people and exclude interventions that involve other caregivers. To further improve the methodological reproducibility and rigor of this scoping review, we will comply with guidance from the Joanna Briggs Institute and PRISMA.

## Supporting information

S1 Checklist PRISMA-P (Preferred Reporting Items for Systematic review and Meta-Analysis Protocols) 2015 checklist: Recommended items to address in systematic review protocol. (DOCX)
